# Comparison of Laparoscopy and Laparotomy in Early-Stage Endometrial Cancer: Early Experiences from a Developing Country

**DOI:** 10.1155/2020/2157520

**Published:** 2020-04-30

**Authors:** Yusuf Cakmak, Duygu Kavak Comert, Isik Sozen, Tufan Oge

**Affiliations:** Department of Gynecologic Oncology, Eskisehir Osmangazi University School of Medicine, Eskisehir, Turkey

## Abstract

After minimally invasive surgery gained popularity in gynecology, laparoscopic operations became widespread among oncologic operations. However, more studies evaluating experiences of oncologic surgeons during the learning period of laparoscopy are needed. To compare the surgical outcomes and perioperative complications of laparoscopic surgery and laparotomy in the treatment of early-stage endometrioid endometrial cancer patients, we retrospectively investigated patients who underwent surgery due to endometrial cancer at our institution between 2014 and 2018. Early-stage (stage I) endometrioid endometrial cancer patients were included in the study. Operative times, length of hospital stay, extracted pelvic lymph nodes, perioperative complications, and blood loss were compared. A total of 128 patients were treated for stage I endometrial cancer during the study period. Sixty-two patients (48.4%) underwent laparoscopic surgery, and 66 (51.6%) patients underwent laparotomy. Median operation time and pelvic lymph node count in the laparotomy and laparoscopy groups did not demonstrate statistically significant differences. However, the length of hospital stay, estimated blood loss, and perioperative complication rate were lower in the laparoscopic surgery group. Laparoscopic surgery in early-stage endometrial cancer may be performed with less blood loss, shorter duration of hospital stays, and similar lymph node counts compared to laparotomic surgery.

## 1. Introduction

Endometrial cancer (EC) is the most common type of gynecological malignancy among women in developed countries [1] and the second most common in developing countries [2]. Nearly 75% of patients were diagnosed while the disease was confined to the uterus. The endometrioid type of EC is the most common histologic subtype and has a generally favorable prognosis. The standard treatment for this pathology is surgical staging, including total hysterectomy, bilateral salpingo-oophorectomy, and dissection of pelvic and paraaortic lymph nodes for presumed early-stage disease; there is an ongoing debate about whether to perform surgery with or without pelvic and paraaortic lymphadenectomy [3]. Surgery is the mainstay of management of endometrial cancer all around the world.

After minimally invasive surgery gained popularity in gynecology [4], laparoscopic operations became widespread among oncologic operations [5, 6]. Randomized, controlled trials and meta-analysis have demonstrated significantly less morbidity, shorter hospitalization, less pain, and quicker recovery with laparoscopic staging of endometrial cancer [7–12]. Studies evaluating oncologic outcomes including rates of recurrence, disease-free survival (DFS), and overall survival demonstrated noninferiority of laparoscopy for patients with early-stage endometrial cancer [12–14]. Endoscopal practice, especially in gynecologic oncological operations, requires a relatively long learning period and considerable experience [15]. More studies are required which evaluate early experiences of oncologic surgeons during the learning period of laparoscopy.

The advantages of laparoscopic surgery for reducing morbidity and mortality are well-known in high income countries [16]. However, laparoscopic surgery in developing countries is highly variable due to economic reasons and lack of experience [16–18]. Accessing minimally invasive surgery for gynecologic cancer patients could have significant impacts on morbidity, mortality, and quality of life in low- and middle-income countries compared to developed countries [16, 18]. More research from developing countries is required to establish the surgical and oncologic outcomes of the laparoscopic surgery in treatment of gynecologic malignancies.

The aim of the present study is to compare a single center experience of laparoscopic and laparotomic approaches for the treatment of early-stage endometrioid EC in a developing country.

## 2. Materials and Methods

**Figure 1:**
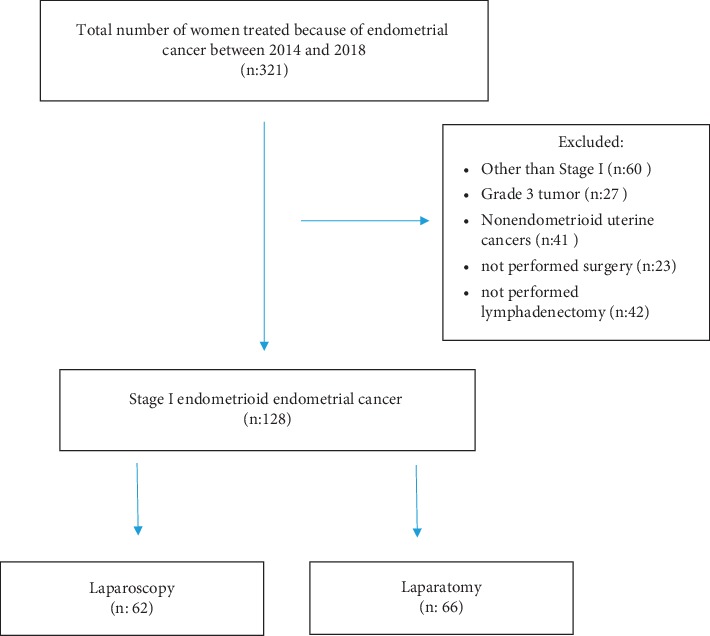
Study population.

For this retrospective study, we reviewed the records of 321 patients who underwent surgical staging due to endometrial cancer in our gynecologic oncology department between 2014 and 2018. Our institution is one of the university hospitals which have been performing laparoscopic surgery for benign gynecologic disorders since 2000. As our laparoscopic settings were upgraded and our experience in laparoscopic surgery improved, laparoscopic surgery for gynecologic cancer has been performed ever since 2013. The local ethics committee approved the study. Diagnosis of endometrial cancer was established by dilatation and curettage. According to histopathologic evaluation, patients diagnosed as early-stage grade I or grade II endometrioid adeno cancer were enrolled in the study group. Stage I grade III endometrioid endometrial cancer patients and types of endometrial cancer other than endometrioid were excluded from the study. Grade III endometrioid adeno cancers and nonendometrioid types are considered to be high risk groups, where systemic pelvic-paraaortic lymphadenectomy to the level of the renal vein is recommended. Laparotomy was performed for all of these patients due to a lack of experience in performing paraaortic lymphadenectomy between inferior mesenteric artery and renal vein in laparoscopic surgery. The study population is presented in Figure 1.

All patients underwent detailed vaginal ultrasonography, discussed in weekly joint gynecologic department meetings. Patients were informed about laparoscopic and laparotomic approach and were surgically staged via laparoscopy or laparotomy. The study's surgeon has performed gynecologic oncology operations via the laparotomic approach for more than 10 years and laparoscopic approaches since 2013. The surgeon evaluated the patients in terms of surgical approach according to patients' preference and feasibility of operation. Laparoscopy or laparotomy was selected according to the surgeon's and patient's preference. All patients underwent total hysterectomy, bilateral salpingo-oophorectomy, cytological sampling, and pelvic lymphadenectomy during operations. The specimens were sent to frozen examination during the operation to examine the myometrial invasion (MI). While lymph node dissection is controversial, paraaortic lymphadenectomy is performed at our institution to the level of the inferior mesenteric artery in patients with greater than 50% MI grade I-II endometrioid adeno cancer; this is because adjuvant therapy after surgery is influenced by the stage of the disease. Fifty-eight patients with greater than 50% MI underwent paraaortic lymphadenectomy. Histological staging and grading were performed according to the current International Federation of Gynecology and Obstetrics (FIGO) classification. Our data included patients' demographic characteristics, clinical parameters including operation time, length of hospital stay, intraoperative and postoperative complications, and final pathology reports from the hospital files.

SPSS for Windows 18.0 and SigmaStat 3.5 were used for data analyses. The normality of distribution was checked initially by Shapiro Wilk's test, and parametric or nonparametric tests were applied to data with normal or nonnormal distributions, respectively. Chi-square tests were applied for categorical variables. Results are expressed as mean standard deviation (SD) and median (interquartile range Q1 and Q3); *p* < 0.05 was considered statistically significant.

## 3. Results

Three hundred and twenty-one patients were treated for endometrial cancer during the study period: 128 of them had stage I grade I or II endometrioid endometrial cancer. One hundred and ninety-three patients were excluded the study (Figure 1). Sixty-two patients (48.4%) underwent laparoscopic surgery, and 66 (51.6%) patients underwent laparotomy. The mean age and body mass index (BMI) of all patients were 61.6 ± 8 and 28.9 ± 6, respectively. The BMI values were slightly higher in the laparotomy group, but this difference was not statistically significant (*p* > 0.05) (Table 1).

The surgical procedure was converted from laparoscopy to laparotomy in four (6.4%) patients due to inadequate Trendelenburg position and vision and severe adhesions. Median operation time and pelvic lymph node count in the laparotomy and laparoscopy groups were not statistically different (*p* > 0.05) (Table 1). However, the length of hospital stay (2.3 days (2–5) vs 5.4 days (4–7)) (*p*=0.02) and estimated blood loss (150 (50–200) cc vs 300 (200–400) cc) (*p*=0.03) were significantly lower in the laparoscopic surgery group. Perioperative complication rate was 6.4% and 16.6% in the laparoscopic surgery and the laparotomy groups, respectively (*p* < 0.05). The list of perioperative complications is shown in Table 2. Survival data were not given due to short follow-up duration time.

## 4. Discussion

This study reveals that laparoscopic surgery, including pelvic lymphadenectomy, has advantages in postoperative recovery including lower blood loss, less ileus, and fewer surgical infections resulting in earlier discharge of the patients from hospital despite the slightly longer operation time. Length of hospital stay was longer in both groups compared to stays observed in developed countries because our institution is one of the reference centers in this region and most of our patients came from neighboring provinces.

Minimally invasive procedures have gained popularity due to their advantages, leading to laparoscopy being used more often in gynecologic oncology [19]. Existing studies evaluating the role of laparoscopy in patients with EC conclude that minimally invasive surgery is feasible and recovery time is quick compared to open procedures [7–15, 20]. In the current study, the average duration of surgery was 110 minutes in the laparotomy group and 120 minutes in the laparoscopy group. In the LAP2 study, the median operative time for laparotomy and laparoscopy was 130 minutes and 204 minutes, respectively [13]. Also, in the LACE study, laparoscopic surgery operation time was found to be statistically significantly longer [7]. Our results differ from two well-known, randomized, controlled trials. The reason for this discordance may be due to the design of the study. Surgeons preferred easier cases during the learning curve and reached similar operation times. Furthermore, laparoscopic operation time in this study begins with the incision of the umbilicus and does not include preparation of the patients, which may alter the results. Evaluation of the study population via BMI also reveals further surgeon-related effects. Patients who underwent laparoscopic surgery were slightly thinner than the others because of the surgeon's anxiety to perform laparoscopy in his/her first cases. Some previous studies have thinner patients who underwent laparoscopy [15, 21], although recent studies have pointed out that laparoscopy can be easily performed on patients with high BMI [19, 22, 23]. After a certain experience level is achieved, high patient BMI should not be considered as a problem. Laparoscopy should be the first choice in order to avoid wound infections in obese women.

Although performing lymph node dissection is an ongoing debate in regards to early-stage EC, pelvic lymph node dissection was performed for all participants in the current study. The number of lymph nodes removed from pelvic area is similar in laparoscopy and laparotomy groups, which is also sufficient for staging based on existing recommendations in the literature [24]. The number of collected pelvic lymph nodes was also similar to that in previous studies [8, 9, 15, 20].

According to the complication rates in the present study, the laparoscopy group demonstrates less febrile morbidity and fewer surgical site, postoperative hernia, ileus, and urinary tract infections. A meta-analysis of RCTs that evaluated pre and postoperative complications concluded that laparoscopy was associated with a significantly lower postoperative complication rate (−6.83%; 95% CI, −9.19% to −4.47%) [10].

The retrospective design of the current study may generate inherent biases including selection and information bias. The study groups were formed mostly according to the clinician's preference. At the outset of the study and due to the clinician's level of experience, laparoscopic pelvic lymphadenectomy patients with lower BMI and less history of previous surgery underwent the laparoscopic procedure. While this creates an obvious selection bias, it has the advantage of permitting evaluation of the initial case results of laparoscopic gynecologic oncology operations from a single surgeon (TO) in comparison to open surgical procedures. The other main limitation of this study is the lack of long-term survival data of the participants. It would be better to compare the results with overall and disease-free survival. However, our study group was inadequate to investigate long-term results.

Although there are randomized control trials and meta-analyses evaluating the role of laparoscopy in the management of EC, more studies are needed to evaluate the initial experiences of surgeons using minimally invasive surgery in gynecologic oncology, especially for young surgeons in developing countries.

## 5. Conclusions

Our data confirmed the role of laparoscopy in the management of early EC for surgeons who are familiar with gynecologic oncology and beginning laparoscopic pelvic lymphadenectomy. Laparoscopic surgery in early-stage endometrial cancer may be performed with less blood loss, shorter duration of hospital stay, and similar lymph node counts to laparotomy and should be supported in endometrial cancer treatment.

## Figures and Tables

**Table 1:** Demographic characteristics of the patients.

	Laparoscopy (*n* = 62)	Laparotomy (*n* = 66)	*p* value
Operation time in minutes, median (range)	120 (100–140)	110 (80–125)	*p* > 0.05
Body mass index (kg/m2 ± standard deviation)	27 ± 4	30 ± 5	*p* > 0.05
Dissected pelvic node count, median (range)	20 (8–28)	22 (12–25)	*p* > 0.05
Intraoperative blood loss (mL)	150 (50–200)	300 (200–400)	**p**=0.03
Days of hospital stay, median (range)	2.3 (2–5)	5.4 (4–7)	**p**=0.02

**Table 2:** Intraoperative and postoperative complications.

	Laparoscopy	Laparotomy
Surgical site infection	—	2
Urinary tract infection	—	1
Paralytic ileus	—	3
Bladder perforation	1	—
Febrile morbidity	—	3
Conversion to laparotomy	4	—
Postoperative hernia	1	2

## Data Availability

The retrospective data used to support the findings of this study are available from the corresponding author upon request.
